# Funduscopy in Adult Zebrafish and Its Application to Isolate Mutant Strains with Ocular Defects

**DOI:** 10.1371/journal.pone.0015427

**Published:** 2010-11-05

**Authors:** Markus Tschopp, Masanari Takamiya, Kara L. Cerveny, Gaia Gestri, Oliver Biehlmaier, Stephen W. Wilson, Uwe Strähle, Stephan C. F. Neuhauss

**Affiliations:** 1 Institute of Molecular Life Sciences, University of Zurich, Winterthurerstrasse, Zurich, Switzerland; 2 Institute of Toxicology and Genetics, Karlsruhe Institute of Technology, Karlsruhe, Germany; 3 Department of Cell and Developmental Biology, University College London, London, United Kingdom; Dalhousie University, Canada

## Abstract

Funduscopy is one of the most commonly used diagnostic tools in the ophthalmic practice, allowing for a ready assessment of pathological changes in the retinal vasculature and the outer retina. This non-invasive technique has so far been rarely used in animal model for ophthalmic diseases, albeit its potential as a screening assay in genetic screens. The zebrafish (*Danio rerio*) is well suited for such genetic screens for ocular alterations. Therefore we developed funduscopy in adult zebrafish and employed it as a screening tool to find alterations in the anterior segment and the fundus of the eye of genetically modified adult animals.

A stereomicroscope with coaxial reflected light illumination was used to obtain fundus color images of the zebrafish. In order to find lens and retinal alterations, a pilot screen of 299 families of the F3 generation of ENU-treated adult zebrafish was carried out.

Images of the fundus of the eye and the anterior segment can be rapidly obtained and be used to identify alterations in genetically modified animals. A number of putative mutants with cataracts, defects in the cornea, eye pigmentation, ocular vessels and retina were identified. This easily implemented method can also be used to obtain fundus images from rodent retinas.

In summary, we present funduscopy as a valuable tool to analyse ocular abnormalities in adult zebrafish and other small animal models. A proof of principle screen identified a number of putative mutants, making funduscopy based screens in zebrafish feasible.

## Introduction

For centuries people wondered what lies behind the pupil. Unaided vision only reveals a black hole while the fundus of the eye itself remains invisible. Only in the 19^th^ century did Helmholtz rationalize that by trying to look into someone else's eye one blocks the light needed to illuminate the eye cavity. Helmholtz overcame this problem by using a transparent mirror made of three thin parallel sheets of glass angled to reflect the maximum light in the eye [1]. This first ophthalmoscope proved to be difficult to use and was soon replaced by a concave mirror with a central opening [2]. This invention revolutionized ophthalmology and nowadays funduscopy is one of its most commonly used diagnostic tools.

Although funduscopy as a non-invasive tool is well established in the clinics, its use to survey ocular alterations in animal model organisms is still not routine. This is particularly true for the zebrafish, a genetic model for vertebrate vision that is becoming increasingly popular [3]. Its retina is cone dominant and a number of disease models of impaired vision exist in larval zebrafish [4], [5], most of which were identified in large-scale mutagenesis screens. The analysis of visual defects in zebrafish larvae is quite advanced, while efforts to analyse the adult visual system is lagging behind. This is arguably one of the reasons why there are only few adult zebrafish models of eye diseases. We set out to establish a simple, fast tool for the identification and study of altered eyes of adult fish. Funduscopy is well suited for such an approach, since it is rapid and non-invasive. It is not only useful to assay for retinal alterations, but also alterations of the optical system (e.g. lens and cornea). Being non-invasive, it is suited to follow the progression of a disease in a single animal or to monitor the impact of a given therapeutic intervention.

To the best of our knowledge, no method to routinely image the fundus of the zebrafish eye has been described in the literature. Instruments designed for humans cannot be used due to the small size of the zebrafish eyes (with a pupil size of around 1 mm [6]). For the mouse eye with a dilated pupil size of 3–4 mm [7] a number of methods have been developed. Fundus images can be acquired by specifically designed fundus cameras [8], by topical endoscopy fundus imaging [7], by counteracting corneal refraction (with a modified Goldmann-type fundus contact lens [9] or a coverslip [10]), and with scanning laser ophthalmoscopy (SLO) [11], [12]. The main obstacle to apply these methods to zebrafish is the even smaller pupil size of the zebrafish eye. In topical endoscopy fundus imaging, the diameter of the endoscope needs to be smaller or equal to the pupil size; the one described for use in mice has an outer diameter of 3 mm [7] and can therefore not be used for zebrafish. Whether SLO (with commercially available instruments) would work in zebrafish is unknown, but again, the laser beam is greater (typically 3 mm in diameter) than the pupil size of the zebrafish. Even if it were possible to use SLO in zebrafish, it has the drawback of not providing color images like funduscopy (but an advantage of SLO is to select lasers of different wavelength to use for fluorescein angiography).

We report here that a stereomicroscope with “coaxial reflected light” or near-vertical illumination can be used for funduscopy in fish (and other small animals). The described setup is inexpensive and well suited to screen adult zebrafish for retinal alterations. In a pilot screen we demonstrate that anterior segment and fundus alterations can be found in living adult zebrafish carrying novel (induced) genetic mutations.

## Materials and Methods

### Animals

Adult wild type zebrafish (*Danio rerio*) of the WIK and Tü strain were used for normal funduscopy. They were bred and crossed as previously described [13]. To test our setup for other animals than zebrafish, one wildtype mouse (Bl/6, female, 8 weeks) was used. All examinations were performed in accordance with the ARVO Statement for the Use of Animals in Ophthalmic and Vision Research and were approved by the local authorities (Veterinäramt Zürich TV4206).

### Experimental Setup and Procedure

**Figure 1:**
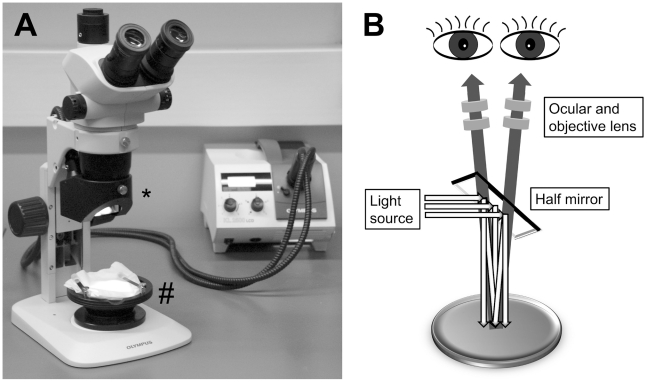
The binocular microscope used for funduscopy. (A) Photograph of the setup; (*) indicates the “coaxial reflected light illuminator” and (#) indicates the ball-and-socket stage. (B) Schematic drawing of the light path.

Of the tested stereomicroscopes, the Olympus SZ-61 equipped with a “coaxial reflected light illuminator” (Olympus, SZ2-ILLC) was found to provide the best images and was therefore used in this study. The “coaxial reflected light illuminator” consists of a half reflecting mirror, a polarizer and a 1/4λ plate. The setup is shown in Figure 1.

For funduscopy, fish were anaesthetized in a solution of 0.014% ethyl-m-aminobenzoate metanesulphonate (MESAB; Sigma) in housing water, taken out of the solution and placed on a wet paper towel. A ball-and-socket stage facilitated the rotation of the fish, which is necessary to see different parts of the retina. To reduce the refraction power of the fish eye, a cover slip or a small concave lens (R = 2.51 mm, diameter 3.5 mm̧ 77.004.41-520, FISBA Optik, St.Gallen, Switzerland,) was placed on the eye. 3% methylcellulose or lubricant eye gel (Viscotears, Novartis) was used to bridge the gap between the cornea and the cover slide, or small lens respectively. Color images of the retina were taken with the camera of the stereomicroscope (Olympus ColorView III or Leica DFC 300FX). As the camera takes square pictures and the magnification of the stereomicroscope is not large enough to fill the whole picture with the image of the fundus, interesting parts of the picture were clipped out using Adobe Photoshop. Magnification has been estimated by magnification of individual lenses and calibrated using known blood vessel thickness.

Mouse funduscopy was essentially the same with the exception that the animal was anesthetized by intraperitoneal injection of Hypnorm (30 µl/20 g body weight; Janssen Pharmaceutics, Beerse, Belgium) and Dormicum (60 µl/20 g body weight; Roche Pharmaceuticals, Basel, Switzerland) diluted with 210 µl Aqua ad inj. (Fresenius Kabi GmbH, Bad Homburg, Germany). To enlarge the pupil of the mouse, 1% atropine was applied on the eye. Again, a cover slide helped to improve image quality.

### Pilot Screen

To find lens and retinal alterations, 299 families (8 fish per family, one eye per fish) of the F3 generation of ENU-treated fish were examined. These fish, aged one year and three months, were part of the European Community ZF-models project (see www.zf-models.org).

### Histology

The enucleated eyes of paraformaldehyde-fixed fish (4% paraformaldehyde in 0.2 M phosphate buffer, pH 7.4, for about one week at 4°C) were dehydrated in a graded series of ethanol-water mixtures, then incubated twice in 1∶1 ethanol and basic solution (Technovit 7100; Heraeus Kulzer, Hanau, Germany) for 1 hour. After overnight infiltration in basic solution, larvae were positioned in polymerization medium (Technovit 7100; Heraeus Kulzer) overnight at room temperature.

Microtome sections (3 µm) were prepared and mounted on slides (Menzel-Gläser, Braunschweig, Germany), air dried at 60°C, stained with modified Richardson solution (0.25% methylene blue, 0.25% borax, and 0.5% azure II in ddH_2_O), overlaid with rapid mounting medium (Entellan; Merck, Darmstadt, Germany), and cover-slipped.

## Results

### Funduscopy in Zebrafish

After testing different illuminations, we found “coaxial reflected light” illumination gave the highest quality images of the zebrafish eye. Near-vertical illumination of operating microscopes also gave good results, although the magnification of the tested operating microscopes was rather small. Coaxial illumination (not reflected) is similar to near vertical illumination and should therefore also be suitable for funduscopy. However, the tested binoculars with coaxial illumination gave dark and dull pictures, likely due to the strong polarizers that are usually used with coaxial illumination to reduce reflection.

Both cover slips and small concave lenses can be used to reduce the refraction power of the fish eye by directly applying them on the cornea. The field of view obtained when using concave lenses is slightly larger compared to that obtained with cover slides. Although a large field of view is favourable when screening, it is more convenient to use cover slides since they are easier to handle and disposable.

### Application of the Funduscopy Setup on Assessing Anterior Eye Segments

The cornea and lens can be examined with standard illumination (other than coaxial or near vertical illumination). However, since light from standard illumination does not travel to the posterior part of the eye chamber, it does not allow viewing of the entire lens (in zebrafish, the lens extends almost to the retina). With coaxial or near-vertical illumination, the entire lens can be examined. Hence our funduscopy setup can be employed for biomicroscopy of the anterior segment. This may help to discriminate different forms of cataract and to find alterations in the posterior part of the lens.

**Figure 2:**
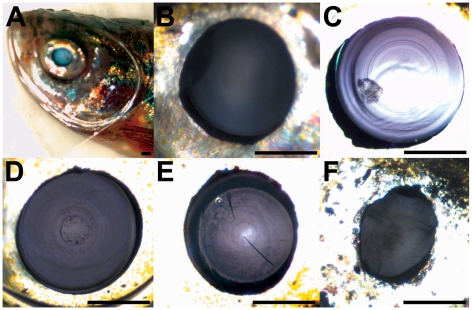
Images of zebrafish eyes. (A) Overview of anaesthetized fish with cover glass, (B) shows a normal, transparent lens, (C–F) examples of opaque lenses (cataracts): (C) small inclusion are present in an otherwise normal lens, (D) massive cataract, (E) including cracks in the lens, and (F) membranous cataract. Scale bar: 500 µm.

In adult zebrafish (about 15 months old) of the ZF-models screening project, we found cataracts in 52 of 299 screened families (on average in 2.5 of 8 fish screened per family). Cataracts can be grouped according to the affected structure. Although the morphogenesis of the lens in zebrafish shows important differences to that in mammals, the overall morphology of the adult zebrafish lens is similar to that of other vertebrates [6]. In principle, each of the lens structures (cortex, nucleus, embryonic lens nucleus) can become opaque and therefore cause a cataract. Indeed, we encountered cataracts of all these lens structures; and combinations of them. Like in humans, the degree and appearance of cataract is highly variable. Some fish showed only a mild form with small inclusions (Fig. 2C), others showed a massive cataract (Fig. 2D–2F), sometimes with cracks in the lens (Fig. 2E), some even showed a membranous cataract Fig. 2F (similar to some forms of congenital cataracts in humans). Interestingly, we also isolated one family in which a body shape reminiscent of scoliosis cosegregated with the presence of cataracts (cataract depicted in Fig. 2D). A number of rare congenital human diseases show this combination of syndromes, which are often caused by chromosomal abnormalities (e.g. chromosome 4 ring, chromosome 6 monosomy, and trisomy 8 syndromes). This putative mutation might be dominantly inherited, since we found 4 affected fish in one family of 8 surveyed individuals.

### Application for Spotting Retinal Alterations

**Figure 3:**
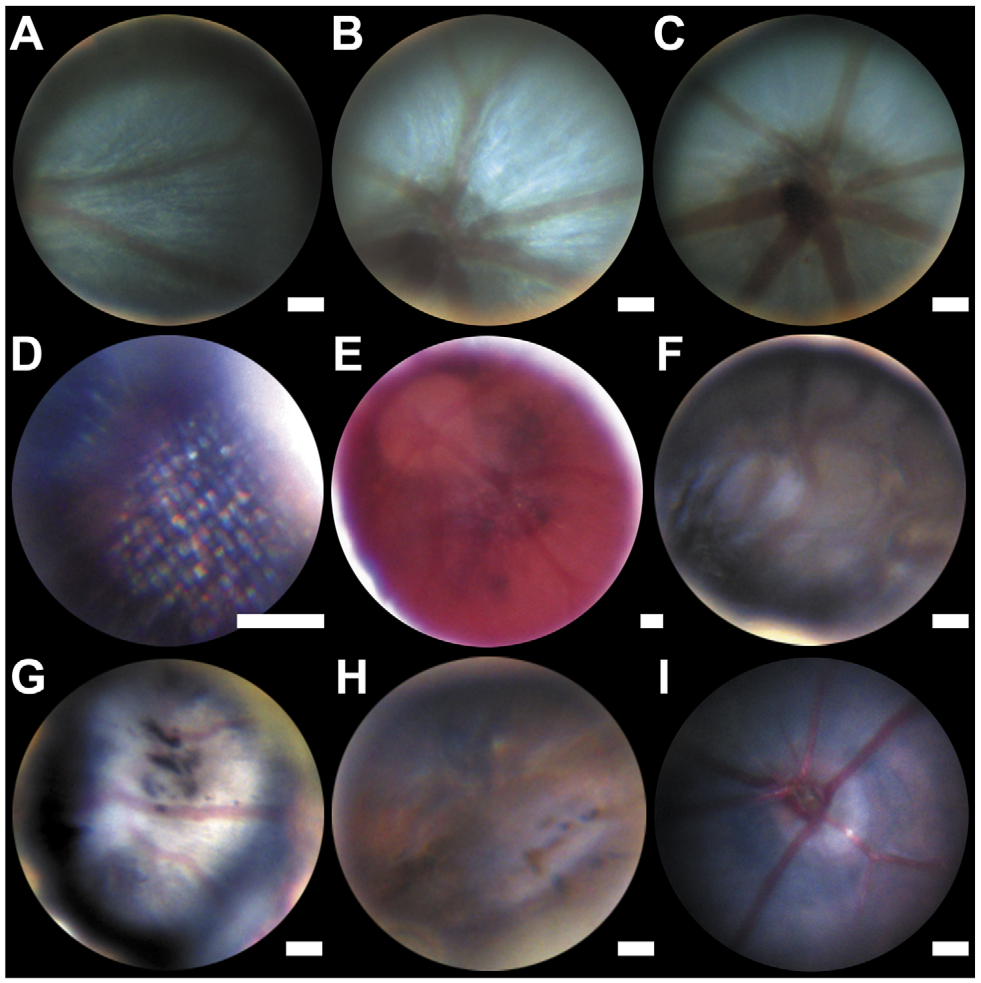
Zebrafish and mouse fundus images. (A–D) wild type zebrafish fundus at the level of the mid-peripheral retina (A), optic disc (B and C) and photoreceptor level, showing the presumed photoreceptor mosaic (D). Hypopigmented fundus of the zebrafish *albino* strain (E). Tortuous arteries and a darkened retina (F), black spots are visible in the retina (G and H). Fundus Image of a wild type mouse (I). Estimated scale bar: 50 µm.

Our setup allows the examination of the retina, retinal blood vessels, and the optic disc in great detail. Due to the high magnification, it is not possible to have all layers of the retina simultaneously in focus. Focusing on the inner part of the retina, also images retinal blood vessels (Fig. 3 A–C). By focusing further outwards, a regular pattern becomes visible (Fig. 3D). This pattern may very well reflect the photoreceptor mosaic typically found in teleost fish [3].

Hypopigmentation of the eye is readily apparent, as illustrated in the zebrafish *albino* strain (Fig. 3E).

The assessment of altered retinal blood vessels is important, since blood vessel alterations are associated with many retinal and systemic diseases including diabetes mellitus and hypertension [14], [15]. In humans and mice, the retina contains arteries and veins (entering and leaving, respectively, the eye at the optic disc). The types of blood vessel can be discriminated by funduscopy. In zebrafish, there are only arteries (in most cases 6–7) leaving the optic disc [16] which arborise radially from the optic disc and eventually anastomose with neighbouring capillaries before connecting to a circumferential vein. This vein circumscribes the retina at the ciliary marginal zone [16]. In accordance with these findings, we typically observed around 7 arteries leaving the optic disc (Fig. 3 C). The circumferential vein cannot be seen with our setup due to its very peripheral location. In healthy animals, retinal arteries proceed in a nearly linear way. We found four families with severe tortuosities (abnormal winding) of blood vessels (Fig. 3F). This demonstrates that blood vessel alterations can be reliably detected with our setup.

**Figure 4:**
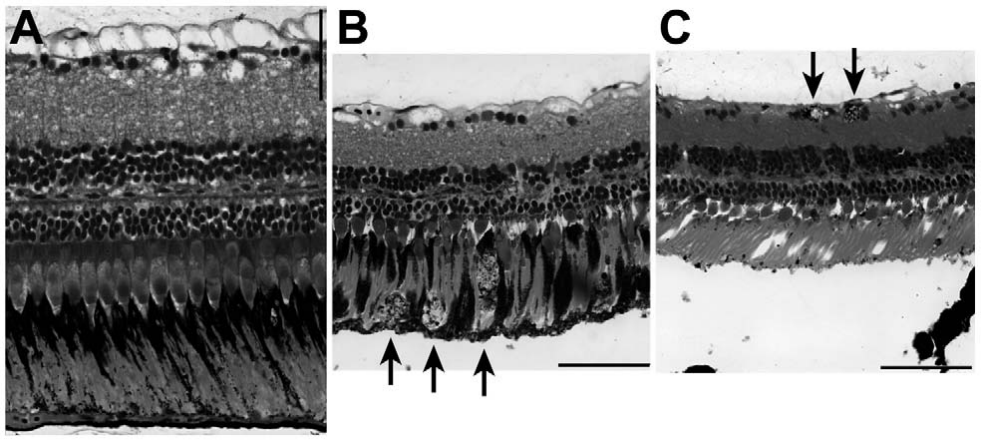
Adult retina sections. (A) Wild type zebrafish. (B) Right eye and (C) left eye of a fish with black spots in the retina; the funduscopy of this fish is shown in Figure 3 (H). Arrows indicate cells with black granules: likely macrophages filled with RPE cells or detached RPE cells. The detached RPE in (C) is a histological artefact. Scale bar: 50 µm.

Additionally, we found retinal alterations in three families with features analogous to mammalian retinal degenerations. One family (9 of 32 fish) showed tortuous arteries and a darkened, somehow bumpy or uneven retina (see Fig. 3F). In another family, in one fish (of 34 tested) black spots appeared on the inner retina (see Fig. 3G). Due to the low incidence, this case falls outside of our criterion of a genetic mutation, but demonstrates that such phenotypes can in principle be identified by funduscopy. In the third family, 3 of 10 tested fish showed small dark spots, which were sometimes arranged in lines (see Fig. 3H). To verify the location of these spots, we sacrificed one fish for histology. Indeed, the black spots seen in funduscopy correlated well with an accumulation of pigmented cells in the inner plexiform layer and may be similar to the bone-spicule bodies that are found in the eyes of patients with retinitis pigmentosa (Fig. 4). As in retinitis pigmentosa, these cells are likely to be macrophages that have phagocytosed retinal pigment epithelium (RPE) cells or RPE cells that have detached from the RPE layer [17]. In addition to the black cells in the inner retina, the outer retina showed massive photoreceptor degeneration and the same black cells in the RPE layer.

In the diseased human retina, the most noticeable retinal changes besides blood vessel alterations and bone-spicule like bodies (e.g. in retinitis pigmentosa), are drusen (e.g. in age related macular degeneration). Drusen are conspicuous extracellular accumulations at the level of Bruch's membrane. In our screened fish, we did not find any evidence for drusen.

### Application to Other Animals

The described setup should work for all animals that can be placed below a stereomicroscope. As a cursory proof of principle, we tested the setup on one wild type mouse (Fig. 3I). The obtained image quality is comparable to the one described by Hawes et al [8] who used a fundus camera applying the principle of indirect funduscopy. Image quality was compromised by breathing movements of the animal, which can be readily ameliorated with more sophisticated anesthesia.

## Discussion

Funduscopy is one of the most important tools in ophthalmology, and will be of growing importance in the study of animal models of ocular diseases. It already proved its usefulness in mice research where it is routinely applied. For instance, this technique was applied to isolate strains with spontaneous [8], [18], [19] or chemically induced [20] mutations leading to heritable eye mutations and to study acute toxic effects on the eye [18]. Being non-invasive, it shows its power in characterizing and monitoring retinal phenotypes over time in the living animal [21], [22], [23].

In this paper we describe a simple and cheap method to obtain fundus images of adult zebrafish. The zebrafish model system is increasingly popular to study ophthalmic diseases [3]. However most studies to date focus on larval stages, due to the availability of visually impaired mutant strains and the amenability of larval stages to gene knockdowns. In the future, genetic manipulations leading to alterations of the adult visual system will be increasingly used. Funduscopy is an ideal method to non-invasively assay eye alterations. This is of importance to preserve mutations with dominant effects, since assayed fish are needed for further breeding. Since many human eye diseases are progressive and manifest at advance age, adult zebrafish have the potential of being more appropriate models for such diseases as age related macular degeneration than larval fish.

We applied the principle of direct ophthalmoscopy, based on the use of the subject's lens as magnification device. Since the refractive power of aquatic lenses is higher than those of terrestrial animals, we obtain a much larger magnification than in the human eye. Therefore the magnification is easily sufficient to image blood vessels and even large enough to image the presumed photoreceptor mosaic of the retina (Fig. 3D). High magnification is often desirable, however the drawback is that it limits the field of view. In our set-up we do not see the whole fundus in a single image. In order to screen the whole fundus, we therefore have to move the specimen, which enabled us to screen fairly rapidly. One eye takes about half a minute to evaluate. The images in Figure 3 represent about 5% of the entire fundus. The use of this technique is not limited to zebrafish, but is applicable to all animals with small eyes, as we have demonstrated by obtaining the fundus image of a mouse (Fig. 3I).

To demonstrate the feasibility of zebrafish funduscopy for the isolation of adult fish strains with morphological eye alterations, we screened 299 lines of F3 adult zebrafish descended from chemically mutagenized founder fish. For each fish line, we screened at least 8 fishes and only phenotypes that were seen at least twice were considered to be potential mutants. Since this is a pilot proof-of-principle screen, we did not prove the heritability of these traits. However all described phenotpypes appear at least twice per family, and are therefore in the range of expected Mendelian ratio for recessive mutations. In few cases we found half of the assayed fish to be affected, suggesting dominant inheritance.

A total of 59 potential mutants were found, with the great majority (n = 52) displaying lens defects. This finding is in line with clinical data, considering the high incidence of cataracts in elderly humans (almost 50% in persons aged 75 years and more) [24]. At this point it is not possible to clearly assign these lens alterations to a heritable or environmental cause. In future studies it will be interesting to determine the frequency of cataracts in aged wild type fish. We expect a substantial age dependent incidence of lens alterations even in wild type. The high frequency of opaque lenses in adult zebrafish certainly needs to be taken into account when measuring visual performance in behavioral experiments.

Four families displayed aberrations of the retinal vasculature in that the arteries appear abnormally twisted (tortuous). In humans, many disease classes may produce tortuosity, including high blood flow, angiogenesis and blood vessel congestion [25].

Three families displayed retinal alterations. Some of these alterations resemble the bone-spicule-like degeneration seen in retinitis pigmentosa, both in funduscopy and in subsequent histological sections (Fig. 3H and Fig. 4). Retinitis pigmentosa is a human disease that usually starts in adulthood, therefore such adult (putative) mutants are of particular interest since they may provide a closer resemblance to human retinal degeneration than the available larval models [3]. They also provide the opportunity to identify genes implicated in disease that when mutated do not exhibit embryonic of larval phenotypes. Although some adult zebrafish models with late-onset photoreceptor cell degeneration exist [26], [27], the characteristic bone-spicule like degeneration has not previously been described. In the limited cohorts examined, we found no mutant fish with drusen-like alterations, which in humans are associated with age-related macular degeneration [28]. Since we only screened a limited number of mutant lines, more extensive screens may very well uncover lines with such alterations in the outer retina.

In summary, we have developed a simple and fast method to obtain fundus images of adult living zebrafish. This is the first report of funduscopy in zebrafish and its use in isolating adult ocular mutants. This procedure is simple and fast enough to be useful for forward genetics screens, as we have demonstrated in a pilot genetic screen, which identified several interesting fish lines with ocular alterations, affecting lens, blood vessels and outer retina. Since we have not demonstrated the heritable nature of these alterations, we can only assume them to be genetic mutations due to their approximate Mendelian ratio. The successful isolation of such fish in a screen based on funduscopy demonstrates that such screens are possible and are expected to lead to the isolation of interesting mutant lines, relevant as disease models of age related eye diseases.
